# Optimization of Bioprocess Extraction of *Poria cocos* Polysaccharide (PCP) with *Aspergillus niger* β-Glucanase and the Evaluation of PCP Antioxidant Property

**DOI:** 10.3390/molecules25245930

**Published:** 2020-12-15

**Authors:** Peng Wu, Hongyuan Tan, Jianfeng Zhan, Weixin Wang, Ting Hu, Shiming Li

**Affiliations:** 1 Hubei Key Laboratory of Economic Forest Germplasm Improvement and Resources Comprehensive Utilization, Hubei Collaborative Innovation Center for the Characteristic Resources Exploitation of Dabie Mountain, Hubei Zhongke Industrial Technology Research Institute, Huanggang Normal University, Huanggang 438000, China; wupeng201412@163.com (P.W.); mm08901@yahoo.com (H.T.); zhanjianfeng2010@163.com (J.Z.); wangweixin1009@aliyun.com (W.W.); 2 Department of Food Science, Rutgers University, New Brunswick, NJ 07102, USA

**Keywords:** *Poria cocos* polysaccharide, biotransformation, β-glucanase, optimal processing conditions, response surface method, antioxidant activity

## Abstract

*Poria cocos* mushroom is widely used as a food and an herb in East Asian and other countries due to its high nutritional value. Research has demonstrated that *Poria cocos* polysaccharides (PCP) are the major bioactives and possess antioxidation, anti-inflammation, immunoregulation, and other health promoting properties. However, the efficient preparation of PCP has been a challenge, particularly in large scale for industry. Herein, we investigated the biotransformation of PCP from *Poria cocos*, catalyzed by β-glucanase from *Aspergillus niger* and focused on optimizing the most four influencing parameters: Temperature, time, pH, and enzyme dosage in this study. After numerous optimizations with the assistance of response surface optimization methodology, we have established that the optimal conditions for the biotransformation PCP preparation were as following: Enzymolysis temperature 60 °C, time 120 min, pH 5.0 and enzyme dose 20 mL. Under these conditions, the extraction yield of PCP reached as high as 12.8%. In addition, the antioxidant activities of PCP were evaluated by reducing power assay and 1,1-diphenyl-2-picryl-hydrazyl, superoxide anion, and hydroxyl radicals scavenging assays. Resulting data showed that PCP presented outstanding antioxidant capacity. Thus, these findings indicate that PCP could be produced as a natural antioxidant for further development.

## 1. Introduction

*Poria cocos* (*P. cocos*), also called Fu-Ling in Chinese, is widely used as a food or food supplement and an herb medicine in china and other East Asian countries due to its health promoting effects [1,2,3]. The countries that produce *P*. *cocos* were Japan, North Korea, North America, Australia, and China. It is traditionally used to treat chronic gastritis, nephrosis, dizziness, nausea, edema, catarrh, and emesis, among others [2,4]. *P. cocos* have various constituents, including polysaccharides [5], triterpenes, steroids [6,7], and small molecular constituents [8]. Among them, polysaccharide is the main active ingredients and accounts for approximately 93% of dry sclerotia of *P. cocos* [5,9]. Studies in recent years have demonstrated that polysaccharides from *P. cocos* (PCP) possess various biological activities, including antioxidation [10,11,12], inhibition of inflammation [13], immunoregulation [14], anti-tumor [4,15], anti-proliferation [11], lowering effects of hyperglycemia and hyperlipidemia [16], anti-depression and hepatoprotective effects [17]. Recently, polysaccharides from *P. cocos* have been recognized as safe and effective antioxidants. Compared with other synthetic antioxidants, PCP have no potential hazards to health, such as carcinogenesis and liver damage [17]. Thus, it is essential to study the extraction technology of PCP for the subsequent antioxidation experiments.

At present, the preparation method for PCP was mainly hot water extraction [1]. The disadvantages of this extraction method are long duration, poor efficiency, and low yield [1,18]. Hence, other methods were explored to extract PCP, such as ultrasonic-assisted extraction, microwave-assisted extraction, and enzyme catalyzed extraction [9,11,19]. The structure of the polysaccharides extracted by ultrasound and microwave is often destroyed resulting in unpredicted loss of biological activity. Enzyme assisted extraction is undoubtedly an emerging technology in the food industry [7,20]. Enzyme extraction technology has proved to have many advantages than conventional techniques in sustainable extraction of polysaccharides, such as improved pharmacological activity of the extract [21], high extraction yield, lower investment costs and energy requirements, high reproducibility at shorter times, simplified manipulation, and more green [7,22]. However, the enzymes employed in previous extraction is very expensive [23], thus these enzymatic methods are impractical or not applicable for massive production. In this study, we aimed to explore an optimized enzymatic process that is both efficient and affordable to be potentially used in large scale extraction of PCP from *P. cocos,* and also to detect the antioxidant property of isolated PCP as a preliminary quality control. Hence, we isolated a β-glucanase from fermentated *Aspergillus niger* (*A. niger*) HS-5 that was used to assist the PCP extraction from *P. cocos* mushroom. To obtain the optimized enzymatic extraction conditions of PCP, we employed four single factor experiment (enzymolysis temperature, initial pH, enzymolysis time, and enzyme dose) and response surface methodology (RSM). We further evaluated the antioxidant activity of PCP by measuring the total antioxidant activity and the scavenging capacity of 1,1-diphenyl-2-picryl-hydrazyl (DPPH), superoxide anion and hydroxyl radicals. The work laid the foundation for the extraction conditions used in mass production and the development of PCP as a functional food.

## 2. Results and Discussion

### 2.1. Preparation of A. niger HS-5 β-Glucanase

#### 2.1.1. Organism and Culture Conditions

*A. niger* HS-5 used in this study was isolated from the yeast produced from a local traditional distiller (Macheng distiller Inc., Macheng city, China). The fungus was maintained on multiple potato dextrose agar plates until use. For enzyme production, *A. niger* was grown in optimum liquid medium: (g/L. bran, 50; yeast extract, 3.0; KCl, 0.2; CaCl_2_ 0.6; pH 5.0). The *A. niger* culture medium was incubated at 28 °C on an orbital shaker (150 rpm) for 7 d, then was filtered and harvested. The filtrate was combined as the crude for β-glucosidase preparation.

The enzyme was purified with ammonium sulfate salting out method at 4 °C, namely, saturated ammonium sulfate was added to the crude filtrate containing β-glucosidase and resulted precipitate was collected by centrifugation, which was subsequently dialysized with distilled water. Further purification was performed via gel filtration on a Sephadex G-100 chromatography column [24].

#### 2.1.2. Enzyme Assay

β-glucosidase activity in the crude culture filtrate and different fractions was determined in assay mixtures containing p-nitrophenyl β-d-glucopyranoside (Merck Inc., Kenneworth, NJ, USA) at 5 mM in 0.05 M of citrate buffer (pH 5.0) and aliquots of diluted enzyme source with controls. The reaction was terminated after incubation for 15 min at 45 °C, by adding 2 mL of 0.2 M sodium carbonate. The production of p-nitrophenol was determined in a Spectronic-73 spectrophotometer (Spectrum Instruments, Shanghai, China) at 410 nm. All assays were performed in three duplicates. The β-glucosidase activity was one unit when the amount of enzyme consumed to release one micromole of p-nitrophenol per minute under assay conditions [24].

### 2.2. Effects of Four Single Factors on Extraction Yield of PCP

#### 2.2.1. Enzymolysis Temperature

**Figure 1:**
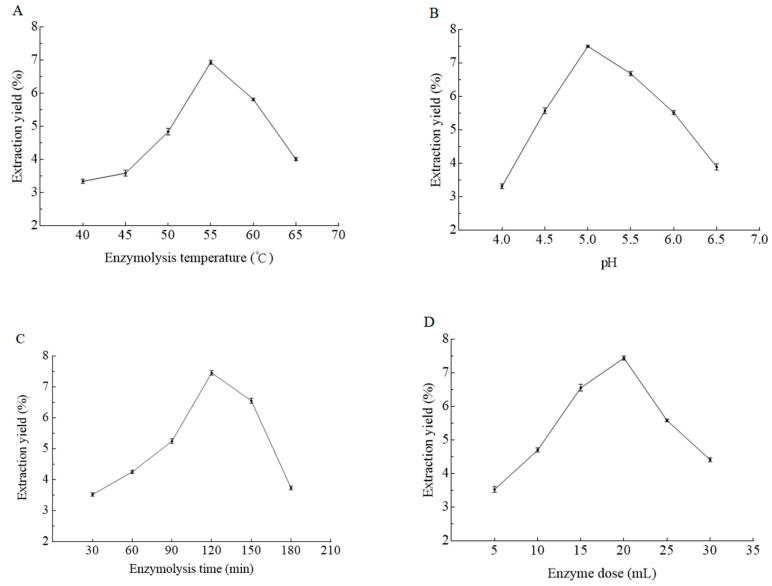
Effects of enzymolysis temperature (**A**), pH (**B**), enzymolysis time (**C**), and enzyme dose (**D**) on extraction yield of *Poria cocos* polysaccharides (PCP).

Different enzymes have their own working temperature. To investigate the effect of different enzymatic reaction temperature on the extraction yield of PCP, various temperatures (40, 45, 50, 55, 60, and 65 °C) were tested with other extraction conditions fixed. As shown in Figure 1A, the extraction yield of PCP increased with elevated extraction temperature and reached the maximum value of 6.94% at 55 °C, and then decreased when the extraction temperature increased from 55 to 65 °C, indicating that the optimal enzymolysis temperature was 55 °C. This tendency was also found in another study [23]. The activity of β-glucanase from *A. niger* increased along with the increase of the enzymolysis temperature from 40 to 55 °C and reached the highest at 55 °C, whereas higher temperature than 55 °C caused the decline of β-glucanase activity, leading to the decrease of PCP extraction yield. The exact temperature around 55 °C for the maximum enzyme activity needs further explored when necessary.

#### 2.2.2. pH Value

The effect of different enzymolysis pH on the extraction yield of PCP is shown in Figure 1B. The extraction yield of PCP increased firstly and then decreased with the increase of pH value, reaching the maximum yield at pH 5, indicating that β-glucanase from *A. niger* was most active at pH 5 for the catalytic reaction. When the pH was lower or higher than this value, the β-glucanase activity decreased and the enzyme reactions were not complete, leading to low extraction yield of PCP. The phenomenon was in agreement with Li et al. [21]. Different enzymes have their own preferred appropriate pH value for maximum activity, the spatial structure of the enzyme altered when the pH was changed, causing the change of enzyme conformation and enzymatic activity [25,26].

#### 2.2.3. Enzymolysis Time

The effect of time in the enzymatic reaction on the extraction yield of PCP is illustrated in Figure 1C. It can be seen that the extraction yield of PCP increased with the extension of reaction time from 30 to 120 min, and then decreased when the time exceeded 120 min. The extraction yield of PCP reached the maximum value of 7.46% when the enzymolysis time was 120 min and decreased thereafter. The reason may be that the longer extraction time could lead to further hydrolyzation of PCP by β-glucanase from *A. niger*.

#### 2.2.4. Enzyme Dosage

The dosage of enzyme is an important factor affecting the extraction efficiency of polysaccharides. As shown in Figure 1D, the extraction yield of PCP increased with the increase of enzyme dose and reached the maximum value of 7.44% when the enzyme dose was 20 mL, then decreased as the extraction proceeded. Due to the enzymatic hydrolysis of β-glucanase from *A. niger*, the network structure of cell wall was destroyed, and PCP was continuously dissolved out. When the enzyme dose continued to increase beyond 20 mL while other factors were kept constant, the extraction yield of PCP decreased. This phenomenon was also reported in Chai’s work [27]. The plausible explanation for this phenomenon was that the enzyme molecules in the solution were saturated and the PCP had been basically dissolved out. The excess amount of enzyme may inhibit its activity, leading to the decrease of PCP yield.

According to the above results, enzymolysis temperature 50, 55, and 60 °C, pH 4.5, 5.0, and 5.5, enzymolysis time 90, 120, and 150 min, enzyme dose 15, 20, and 25 mL were chosen for RSM experiments.

### 2.3. RSM Model Building and Statistical Analysis

On the basis of the above single factor test, a Box–Behnken design (BBD) was used to investigate the effects of enzymolysis time, temperature, pH and enzyme dose on the extraction yield of PCP. The experimental conditions of 29 runs and the extraction yields of PCP were shown in Table 1. The yield of PCP ranged from 6.51% to 12.68%. The quadratic regression fitting equation for the extraction yield of PCP is as follows:

*Y* = 12.04 − 0.49*X*_1_ − 0.38*X*_2_ − 0.27*X*_3_ + 0.47*X*_4_ + 0.015*X*_1_*X*_2_ + 0.16*X*_1_*X*_3_ − 0.79*X*_1_*X*_4_ + 0.73*X*_2_*X*_3_ − 1.86*X*_2_*X*_4_ − 0.32*X*_3_*X*_4_ − 0.98*X*_1_^2^ − 1.76*X*_2_^2^ − 2.46*X*_3_^2^ − 1.68*X*_4_^2^ (1)

where, *Y* is the yield of PCP (%), *X*_1_ is enzymolysis temperature (°C), *X*_2_ is pH value of solvent, *X*_3_ is enzymolysis time (min), and *X*_4_ is enzyme dose (mL).

The results of PCP extraction yield were analyzed by multiple regression analysis. As shown in Table 2, the F value of the model was 15.33 and *p* < 0.0001, indicating that the model was extremely significant [21]. The determination coefficient (*R*^2^) and the adjusted determination coefficient (*R_adj_*^2^) were 0.9388 and 0.8775, respectively, implying that the model has a good fit with the actual yield of PCP extraction yield and can be used to predict the best conditions for extraction of PCP. In addition, the linear coefficient (*X*_1_, *X*_4_) and interaction term (*X*_1_*X*_4_, *X*_2_*X*_3_) was significant (*p* < 0.05), and the interaction term (*X*_2_*X*_4_) and quadratic term coefficients (*X*_1_^2^, *X*_2_^2^, *X*_3_^2^, *X*_4_^2^) was extremely significant (*p* < 0.01). The other terms were considered non-significant (*p* > 0.05).

**Figure 2:**
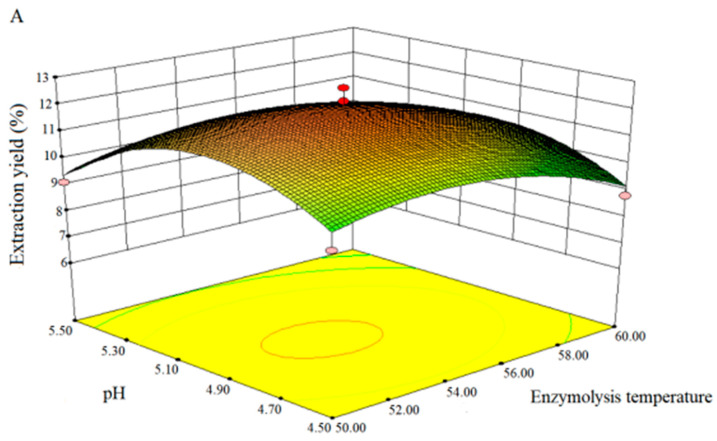

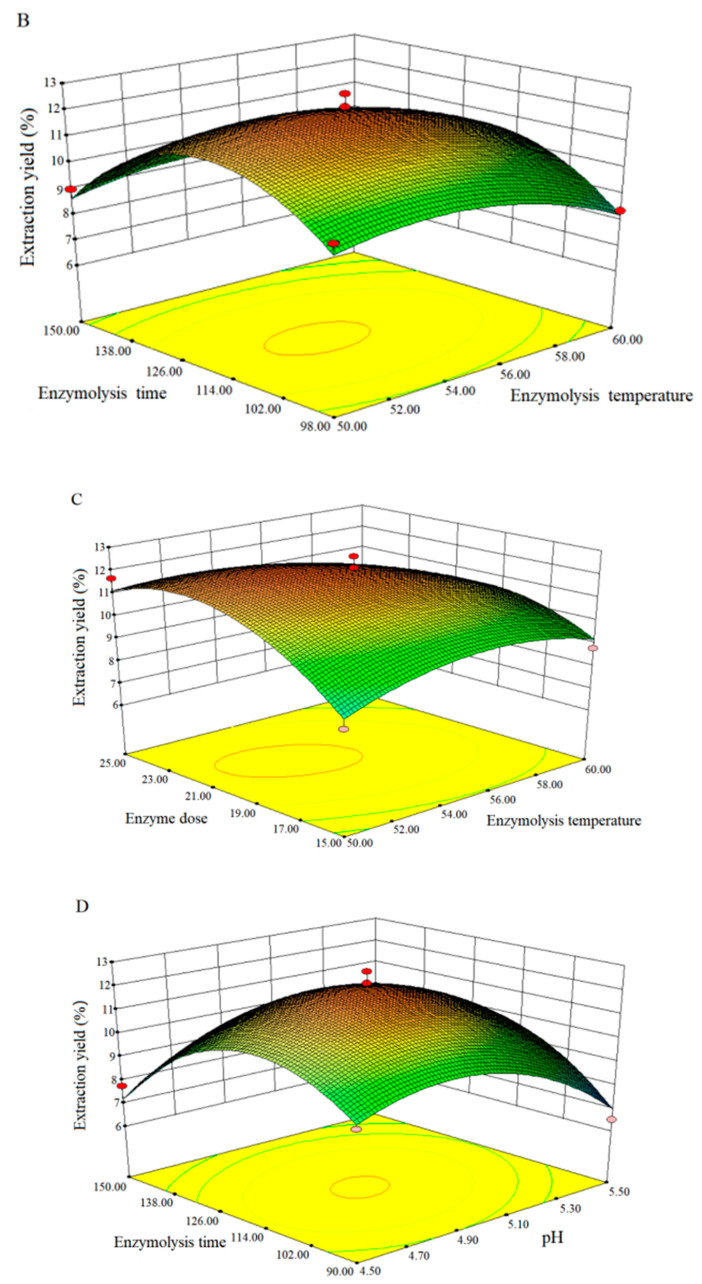

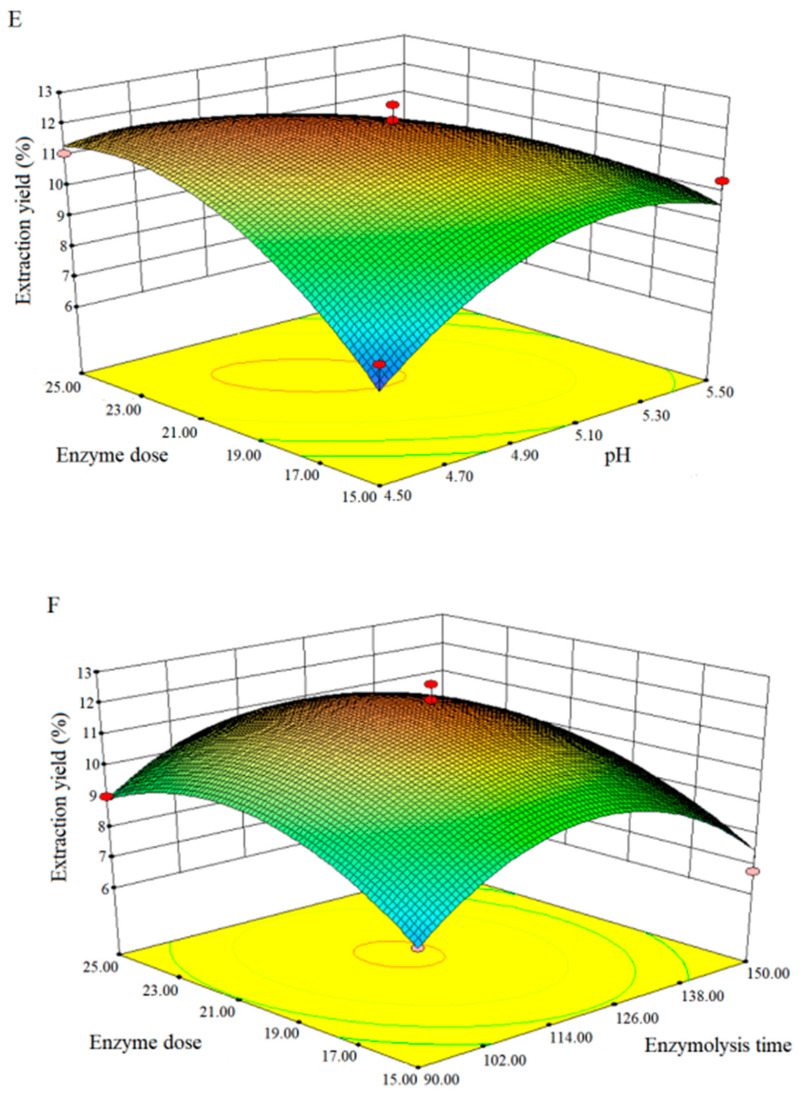
Interaction effects between enzymolysis temperature and pH (**A**), enzymolysis temperature and time (**B**), enzymolysis temperature and enzyme dose (**C**), pH and enzymolysis time (**D**), pH and enzyme dose (**E**), and enzymolysis time and enzyme dose (**F**) on extraction yield of PCP.

We used Design expert 8.0.6 software to draw three-dimensional (3D) response surface to study the effects of parameters and their interaction on the yield of PCP. As shown in Figure 2A–F, each 3D diagram directly exhibited the interaction between the two factors. Figure 2A shows the effect of enzymolysis temperature, pH and their interaction on the yield of PCP at fixed enzymolysis time and enzyme dose (0 level). With the increase of enzymatic reaction temperature, the extraction yield of PCP increased continuously. When the enzymolysis temperature reached a certain value, the extraction yield reached the maximum value, and then showed a downward trend. When enzymolysis temperature was set, the yield of PCP also increased with the increase of solution pH, but when pH exceeded a certain value, the yield of PCP decreased. Too high temperature and pH value would lead to the deactivation of the enzyme and consequently the unwanted hydrolysis of polysaccharide. Likewise, a similar interaction between enzymolysis temperature and time (Figure 2B), enzymolysis temperature and enzyme dose (Figure 2C), pH and enzymolysis time (Figure 2D), pH and enzyme dose (Figure 2E), enzymolysis time and enzyme dose (Figure 2F) on the yield of PCP could be easily obtained. When one of the test variables was a certain value, the yield of PCP increased firstly and then decreased with the changing of another variable.

### 2.4. Model Validation

The predicted optimal extraction conditions from software of Design Expert version 8.0.6 were as follows: Enzymolysis time is 118.77 min, enzymolysis temperature 59.52 °C, pH 4.95, enzyme dose 19.96 mL. The maximum response value predicted by the model was 12.51%. Considering the convenience and feasibility of the actual operation, the modified extraction conditions were as follows: Enzymolysis time 120 min, enzymolysis temperature 60 °C, pH 5.0, and enzyme dose 20 mL. Under these conditions, the average value of PCP extraction yield of thrice experiments was 12.82 ± 0.26%, which was in agreement with the theoretical value (12.51%) predicted by the model, suggesting the prediction result of the model was effective and reliable. It was reported that the microwave-assisted extraction yield of PCP was 9.95% [19], and the ultrasonic-assisted extraction yield of PCP was 1.38% [9]. Thus, the biotransformation extraction yield of PCP was higher than microwave-assisted extraction and ultrasonic-assisted extraction.

### 2.5. Antioxidant Activity of PCP In Vitro

#### 2.5.1. Reducing Power

**Figure 3:**
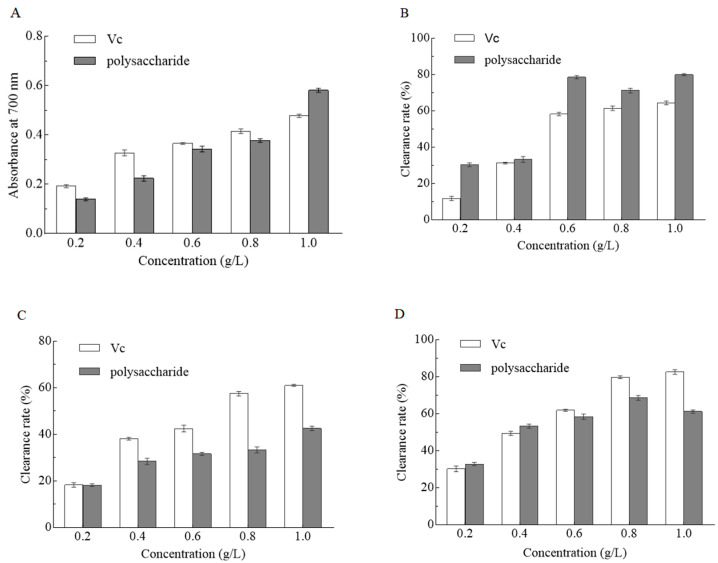
Effect of different concentrations of PCP on the reducing power (**A**), 1,1-diphenyl-2-picryl-hydrazyl (DPPH) radical scavenging effects (**B**), superoxide anion radical scavenging ability (**C**), and hydroxyl radical scavenging ability (**D**).

In this experiment, potassium ferric cyanide reduction method was used, the higher the absorbance, the stronger the reducing ability of polysaccharides [27]. As shown in Figure 3A, the reducing power of PCP exhibited a dose-dependent manner. The absorbance of PCP increased from 0.14 ± 0.005 to 0.58 ± 0.004 in the range of 0.2–1.0 g/L, indicating that the reducing power of PCP had a positive correlation with its concentration.

#### 2.5.2. DPPH Radical Scavenging Activity

DPPH was a free radical compound with a melting point of 127–129 °C, which has a maximum absorption peak at 517 nm [28]. The ethanol solution of DPPH was used to explore the antioxidant capacity of PCP. Under the effect of antioxidant compounds, the delocalized free radical of DPPH combined with a hydrogen atom from the antioxidant, PCP in this case, resulting in the maximum absorption peak disappeared, which was consistent with its principle [29]. As shown in Figure 3B, the DPPH radical scavenging activity was enhanced with the increase of PCP concentration. The maximum value of clearance yield was 79.9 ± 0.8%, much higher than the maximum value of V_C_ (60.4 ± 0.5%) at the same concentration (1.0 g/L), implying that PCP had a better antioxidant activity than that of V_C_.

#### 2.5.3. Superoxide Anion Radical Scavenging Activity

The autoxidation of pyrogallol will be inhibited and its characteristic peak will disappear when antioxidants were added to the experiment [30]. As shown in Figure 3C, in the range of 0.2–1.0 g/L, the clearance rate of PCP increased with the increase of PCP concentration, indicating that the superoxide anion radical scavenging capacity of PCP performed a concentration-dependent manner. Its maximum scavenging rate was 42.9 ± 0.7% at the concentration of 1.0 g/L, suggesting that PCP had potential superoxide anion scavenging activity, but was lower than V_C_.

#### 2.5.4. Hydroxyl Radical Scavenging Activity

In this experiment, *O*-phenanthrene-Fe^2+^ was used as the indicator of oxidation-reduction, and the alternation of reaction can be observed by the change of color. Ascorbic acid (V_C_) is a hydro-soluble antioxidant. As shown in Figure 3D, in the range of 0.2–1.0 g/L of V_C_ concentration, the clearance rate of V_C_ on hydroxyl radical increased with the increase of the V_C_ concentration. In the range of 0.2–0.8 g/L of PCP concentration, the clearance rate of PCP on hydroxyl radical increased with the increase of the PCP concentration. At concentration of 0.8 g/L, the maximum clearance rate reached 68.58 ± 0.9%, which was lower than that of V_C_ (79.77 ± 0.8%). Hence, PCP exhibited a good scavenging ability on hydroxyl radical, although it is not as strong as V_C_.

## 3. Materials and Methods

### 3.1. Materials and Reagents

*Aspergillus niger* HS-5 with high enzyme yield was screened by selective medium and compared its β-glucanase activity. The preparation and isolation of *A. niger* HS-5 β-glucanase was performed in house by using microbial fermentation. In brief, after fermentation, the cells in the fermented broth were removed by centrifugation to obtained crude enzyme solution, then ammonium sulfate salting out method was used to concentrate and Sephadex G-100 chromatography column was used for further purification.

*Poria cocos* were purchased from the Jiuzihe brand (Huanggang, China). Sulfuric acid, phenol, citric acid, sodium citrate, glucose, sodium dihydrogen phosphate, potassium ferricyanide, ferric chloride, trichloroacetic acid, 1,1-diphenyl-2-picryl-hydrazyl (DPPH), anhydrous ethanol, hydrochloric acid, trometamol, pyrogallic acid, salicylic acid, 1,10-phenanthroline, potassium dihydrogen phosphate, dipotassium hydrogenphosphate, sodium chloride, ferrous sulfate, and hydrogen peroxide (30%) were purchased from Sinopharm Chemical Reagent Co., Ltd. (Shanghai, China). All reagents were of analytical grade.

### 3.2. Single Factor Experiment

Two grams of dried *Poria cocos* power was dissolved in 60 mL of buffer solution (pH 5.5, citric acid-sodium citrate) in a conical flask [19]. When the enzymolysis temperature was changed from 40 to 65 °C, other extraction conditions were constant, including initial pH 5.5, enzymolysis time 90 min, and enzyme dose 5 mL. Similarly, when solution pH was changed from 4.0 to 6.5, other parameters were kept constant. The changes of enzymolysis time from 30 to 180 min and enzyme dose from 5 to 30 mL took place alternatively, while other conditions were adjusted accordingly. After the reaction finished, the enzyme was inactivated in boiling water bath for 10 min, and then centrifuged at 3700× *g* for 10 min using high-speed centrifuge (Tg16-wst, Shanghai luXianyi Centrifuge Instrument Co., Ltd.( Shanghai, China) After centrifugation, the supernatant was collected for yield determination of PCP (detailed procedure in next Section 3.3).

### 3.3. Determination of PCP Yield

Improved phenol-sulfuric acid method was employed to determine the content of PCP in the extract [9]. Briefly, 0.25 mL of the extraction solution obtained after centrifugation was mixed with 3.75 mL distilled water in each 25 mL tube, then 2 mL of 5% phenol solution and 6.0 mL of sulfuric acid were added to the mixtures in the tubes. The mixtures were mixed well and kept at room temperature for 30 min. The absorbance value at 490 nm was measured with a UV Visible spectrophotometer (SP-723, Shanghai Spectrophotometer Co., Ltd. (Shanghai, China)). The yield of PCP was calculated by the regression equation of glucose standard curve (y = 0.0108x + 0.0042, R^2^ = 0.999), which was obtained by analyzing a series of standard glucose solutions with concentrations ranging from 0 to 0.02 g/mL.

### 3.4. Response Surface Optimization Experiment

RSM was used to investigate the influence of enzyme dose, time, temperature and pH value on the extraction yield of PCP. A Box and Behnken factorial design, with 4 factors and each varied at 3 levels, was used for the experimental design [9]. The entire experiment was designed in random order, including 5 center point replications and 29 combinations. The coding values and levels of experimental factors of BBD were shown in Table 3.

The variables were coded according to the following formula:

(2) $$x_{i}=\frac{X_{i}-X_{0}}{\Delta X}$$

where *X_i_* is the (dimensionless) coded value of the variable *X*, *X_0_* is the value of *X_i_* at the center point, and Δ*X* is the step change.

The quadratic equation of the influence of experimental factors on response value is as follows:

(3) $$Y=A_{0}+\sum_{i=1}^{4}A_{i}X_{i}+\sum_{i=1}^{4}A_{ii}X_{i}+\sum_{i=1}^{3}\sum_{j=i+1}^{4}A_{ij}X_{i}X_{j}$$

where *Y* is the response variable, *X_i_* and *X_j_* are the independent variables, *A_0_*, *A_i_*, *A_ii_*, and *A_ij_* are the regression coefficients of intercept, linear term, square term and interactive term respectively. Variance analysis was used to evaluate the effect of each independent variable on the response surface, and F-test was used to analyze its significance.

### 3.5. Verification Experiment

The predicted extraction yield of PCP was verified by determining the actual extraction yield under the optimized enzymolysis conditions, and the experiment was repeated three times for reliability analysis [21].

### 3.6. Preparation of Crude PCP

The supernatant was collected under the optimal enzymatic hydrolysis conditions, and anhydrous ethanol was added and kept overnight. The precipitates were collected by centrifugation at 3700× *g* for 10 min, washed with anhydrous ethanol and dissolved in distilled water, then lyophilized to afford extraction samples of crude PCP [11].

### 3.7. Antioxidant Property of PCP

We also investigated the reducing power and the scavenging ability of PCP on hydroxyl free radical, DPPH free radical and superoxide anion free radical.

#### 3.7.1. Reducing Power

The reducing power was assayed according to the reported method with slight modification [31]. Briefly, 2.5 mL of different concentrations of PCP solution in distilled water was mixed with 2.5 mL of phosphate buffer (pH 6.6) and 2.5 mL of potassium ferric cyanide (1.0%, *w*/*v*) in different test tubes. The mixtures were incubated for 20 min at 50 °C. Then, 2.5 mL trichloroacetic acid in water (10%, *w*/*v*) was added to the mixtures and centrifuged at 6165× *g* for 10 min. Five mL of the upper layer was mixed with 5 mL of distilled water and 1 mL of aqueous ferric chloride (FeCl_3_) solution (0.1%, *w*/*v*), and the absorbance was measured at 700 nm. No sample solution was added to the blank control. V_C_ was selected as the control test, and the experiment was performed in triplicate. Reducing power was evaluated employing the following equation.

(4) $$\Delta A=A-A_{0}$$

where A is the absorption value of the sample and A_0_ is the absorption value of the blank group.

#### 3.7.2. Scavenging of DPPH Radicals

The ability of PCP to scavenge DPPH free radicals was determined using the method described by previous literature with some modification [31,32]. Different concentrations of PCP solution were prepared and stored until use. Each of 2 mL PCP solution sample was thoroughly mixed with 2 mL of freshly prepared DPPH and 2 mL of methanol. Then the resulted mixture was incubated at room temperature for 30 min in the dark. The sample absorbance was measured at 517 nm. The scavenging activity of DPPH radicals was calculated according to the following equation:

DPPH scavenging activity (%) = [A_0_ − (Ax − A_x0_)]/A_0_ × 100% (5)

where A_x_ is the absorption value of the sample solution, A_0_ is the absorption value of the blank group determined by replacing the sample solution with anhydrous ethanol, and A_x0_ is the background absorption value determined by mixing the sample with anhydrous ethanol.

#### 3.7.3. Scavenge of Superoxide Anion Radicals

The PCP scavenging ability of superoxide anion radicals was measured using the method described by previous study with modification [28]. Each of 2.5 mL PCP sample at different concentrations in distilled water were mixed with 4.5 mL of Tris-HCl buffer solution (pH 8.2) and incubated at 25 °C water bath for 10 min. Then, 0.1 mL of aqueous pyrogallol solution preheated at 25 °C was added to each of the mixtures. The sample absorbance after 1 min was measured at 320 nm. The scavenging activity of superoxide anion radicals was calculated according to the following equation:

Superoxide anion radical scavenging activity (%) = [(A_0_ − A_x_)/A_0_] × 100% (6)

where A_x_ is the absorbance value measured after adding the sample, A_0_ is the absorbance value of Tris HCl solution as the blank control.

#### 3.7.4. Scavenge of Hydroxyl Radical

The ability of scavenging hydroxyl radicals of PCP was assayed according to the reported method with some modifications [31]. Briefly, 3 mL of different concentrations of PCP in water was mixed with 1.5 mL of Phenanthroline, 3 mL of phosphate buffer and 1 mL of FeSO_4_ solution. Finally, one mL of H_2_O_2_ solution was added in each of the mixtures. The solution was diluted to 10 mL with distilled water and heated in a 37 °C water bath for 1 h. The absorbance of the mixtures was measured at 510 nm. The hydroxyl radical scavenging activity was calculated by the following equation:

Hydroxyl radical scavenging activity (%) = (A_x_ − A_0_)/(A_1_ − A_0_) × 100% (7)

where A_x_ is the absorption value of the test solution, A_1_ is the absorption value of the sample (antioxidant and H_2_O_2_ were not added), A_0_ is the absorption value when H_2_O_2_ is added without antioxidant.

### 3.8. Data Analysis

All data were denoted as mean±standard deviation (SD) of three replicated determinations. The one-way ANOVA analysis and Duncan’s multiple range test (*p* < 0.05) were carried out by SPSS 22.0. The graphs were plotted using Origin 8.0 and GraphPad Prism 5.0 software.

## 4. Conclusions

In this study, we have successfully established the biotransformation and extraction conditions with a potential use in massive extraction of PCP from *P. cocos* with *A. niger* HS-5 β-glucanase. Also we optimized the extraction conditions for the enzymolysis process of PCP by employing response surface method, and found that two factors, i.e., enzymolysis temperature and enzyme dosage, had significantly influenced on the yield of PCP. The actual data obtained under the optimal extraction conditions was in good agreement with the predicted value by the quadratic models. The antioxidant activity of PCP in vitro showed that it has an effective reducing power and a good ability of scavenging on DPPH free radical, superoxide anion and hydroxyl free radical. In summary, the enzymatic extraction method used in this newly found process is mild and high efficiency, and isolated PCP has demonstrated antioxidant function in vitro. Results from this study may provide a novel biotransformation process for PCP extraction in high efficiency and mild conditions, and established a method of further extraction of *P. cocos* polysaccharides to be used in functional foods.

## Figures and Tables

**Table 1:** Box–Behnken design and results.

Standard Number	*X*_1_ Temperature (°C)	*X*_2_ pH	*X*_3_ Time (min)	*X*_4_ Enzyme Dose (mL)	Response Value (%)	
					Measured Value	Predictive Value
1	−1	0	0	−1	8.19	8.61
2	−1	0	0	1	11.66	11.13
3	0	0	0	0	12.20	12.04
4	0	1	0	1	6.55	6.83
5	0	1	−1	0	6.51	6.98
6	1	0	1	0	8.11	7.99
7	0	0	0	0	12.68	12.04
8	0	1	1	0	8.18	7.90
9	−1	1	0	0	9.13	9.40
10	0	1	0	−1	10.37	9.61
11	−1	0	−1	0	9.92	9.52
12	0	0	1	−1	6.76	7.47
13	0	0	0	0	11.45	12.04
14	0	0	0	0	11.93	12.04
15	0	−1	0	−1	7.45	6.65
16	1	0	0	1	9.10	8.57
17	0	0	0	0	11.93	12.04
18	1	−1	0	0	8.81	9.18
19	1	0	0	−1	8.79	9.21
20	0	0	−1	−1	7.37	7.38
21	0	−1	1	0	7.78	7.20
22	0	0	−1	1	9.03	8.95
23	0	−1	−1	0	9.04	9.21
24	1	0	−1	0	8.38	8.22
25	−1	0	1	0	9.01	8.65
26	−1	−1	0	0	9.58	10.19
27	0	0	1	1	7.15	7.78
28	1	1	0	0	8.42	8.45
29	0	−1	0	1	11.08	11.32

**Table 2:** Box–Behnken design variance analysis.

Source	Sum of Squares	df	Mean Square	F Value	*p*-Value Prob > F	Significance
Model	84.95	14	6.07	15.33	<0.0001	**
*X*_1_-Temperature	2.88	1	2.88	7.28	0.0173	*
*X*_2_-pH	1.75	1	1.75	4.42	0.0542	
*X*_3_-Time	0.89	1	0.89	2.24	0.1569	
*X*_4_-Enzyme dose	2.65	1	2.65	6.7	0.0215	*
*X*_1_ *X*_2_	0.9 × 10^−3^	1	0.9 × 10^−3^	2.27 × 10^−3^	0.9626	
*X*_1_ *X*_3_	0.1	1	0.1	0.26	0.619	
*X*_1_ *X*_4_	2.5	1	2.5	6.31	0.0249	*
*X*_2_ *X*_3_	2.15	1	2.15	5.42	0.0354	*
*X*_2_ *X*_4_	13.88	1	13.88	35.05	<0.0001	**
*X*_3_ *X*_4_	0.4	1	0.4	1.02	0.33	
*X*_1_^2^	6.23	1	6.23	15.74	0.0014	**
*X*_2_^2^	19.98	1	19.98	50.48	<0.0001	**
*X*_3_^2^	39.34	1	39.34	99.37	<0.0001	**
*X*_4_^2^	18.31	1	18.31	46.26	<0.0001	**
Residual	5.54	14	0.4			
Lack of Fit	4.73	10	0.47	2.35	0.2134	
Pure Error	0.81	4	0.2			
Cor Total	90.49	28				

* Significant (*p* < 0.05); ** Extremely significant (*p* < 0.01).

**Table 3:** Factors and levels of Box–Behnken design.

Independent Variables	Levels		
	−1	0	1
Enzymolysis temperature (°C) (*X*_1_)	50.00	55.00	60.00
pH (*X*_2_)	4.50	5.00	5.50
Enzymolysis time (min) (*X*_3_)	90.00	120.00	150.00
Enzyme dose (mL) (*X*_4_)	15.00	20.00	25.00

## References

[1] Jia X., Ma L., Li P., Chen M., He C. (2016). Prospects of *Poria cocos* polysaccharides: Isolation process, structural features and bioactivities. Trends Food Sci. Technol..

[2] Sun Y. (2014). Biological activities and potential health benefits of polysaccharides from *Poria cocos* and their derivatives. Int. J. Biol. Macromol..

[3] Wang Y.Z., Zhang J., Zhao Y.L., Li T., Shen T., Li J.Q., Li W.Y., Liu H.G. (2013). Mycology, cultivation, traditional uses, phytochemistry and pharmacology of *Wolfiporia cocos* (Schwein.) Ryvarden et Gilb. J. Ethnopharmacol..

[4] Ke R.D., Lin S.F., Chen Y., Ji C.R., Shu Q.G. (2010). Analysis of chemical composition of polysaccharides from *Poria cocos* Wolf and its anti-tumor activity by NMR spectroscopy. Carbohydr. Polym..

[5] Zhang W.X., Chen L., Li P., Zhao J.Z., Duan J.Y. (2018). Antidepressant and immunosuppressive activities of two polysaccharides from *Poria cocos* (Schw.) Wolf. Int. J. Biol. Macromol..

[6] Lee S., Choi E., Yang S.M., Ryoo R., Moon E., Kim S.H., Kim K.H. (2018). Bioactive compounds from sclerotia extract of *Poria cocos* that control adipocyte and osteoblast differentiation. Bioorg. Chem..

[7] Nadar S.S., Rao P., Rathod V.K. (2018). Enzyme assisted extraction of biomolecules as an approach to novel extraction technology: A review. Food Res. Int..

[8] Liu J., Zhou J., Zhang Q.Q., Zhu M.H., Hua M.L., Xu Y.H. (2019). Monosaccharide analysis and fingerprinting identification of polysaccharides from *Poria cocos* and Polyporus umbellatus by HPLC combined with chemometrics methods. Chin. Herb. Med..

[9] Wang Y., Cheng Z., Mao J., Fan M., Wu X. (2009). Optimization of ultrasonic-assisted extraction process of *Poria cocos* polysaccharides by response surface methodology. Carbohyd. Polym..

[10] Yuan Y., Hua H., Suna X.H., Guan Y., Chen C. (2020). Rapid determination of polysaccharides and antioxidant activity of *Poria cocos* using near-infrared spectroscopy combined with chemometrics. Spectrochim. Acta A.

[11] Chen X., Tang Q., Chen Y., Wang W., Li S. (2010). Simultaneous extraction of polysaccharides from *Poria cocos* by ultrasonic technique and its inhibitory activities against oxidative injury in rats with cervical cancer. Carbohyd. Polym..

[12] Yang H., Wu Y., Gan C., Yue T., Yuan Y. (2016). Characterization and antioxidant activity of a novel polysaccharidefrom Pholidota chinensis Lindl. Antioxidant activity of carboxymethyl (1→3)-β-d-glucan (from the sclerotium of Poria cocos) sulfate (in vitro). Carbohyd. Polym.

[13] Liu X., Wang X., Xu X., Zhang X. (2019). Purification, antitumor and antiinflam-mation activities of an alkali-soluble and carboxymethyl polysaccharide CMP33 from *Poria cocos*. Int. J. Biol. Macrom..

[14] Pu Y., Liu Z., Tian H., Bao Y. (2019). The immunomodulatory effect of *Poria cocos* polysaccharides is mediated by the Ca^2+^/PKC/p38/NF-κB signaling pathway in macrophages. Int. Immunopharmacol..

[15] Huang Q., Jin Y., Zhang L., Cheung P.C.K., Kennedy J.F. (2007). Structure, molecular size and antitumor activities of polysaccharides from *Poria cocos* mycelia produced in fermenter. Carbohyd. Polym..

[16] Sun S.S., Wang K., Ma K., Bao L., Liu H.W. (2019). An insoluble polysaccharide from the sclerotium of Poria cocos improves hyperglycemia, hyperlipidemia and hepatic steatosis in ob/ob mice via modulation of gut microbiota. Chin. J. Nat. Med..

[17] Wu K., Fan J., Huang X., Wu X., Guo C. (2018). Hepatoprotective effects exerted by Poria Cocos polysaccharides against acetaminophen-induced liver injury in mice. Int. J. Biol. Macromol..

[18] Wang Y., Liu S., Yang Z., Zhu Y., Wu Y., Huang J., Mao J. (2011). Oxidation of β-glucan extracted from *Poria Cocos* and its physiological activities. Carbohyd. Polym..

[19] Wang N., Zhang Y., Wang X., Huang X., Fei Y., Yu Y., Shou D. (2016). Antioxidant property of water-soluble polysaccharides from *Poria cocos* Wolf using different extraction methods. Int. J. Biol. Macromol..

[20] Chen H., Zhou X., Zhang J. (2014). Optimization of enzyme assisted extraction of polysaccharides from *Astragalus membranaceus*. Carbohyd. Polym..

[21] Li Y., Zhu C.P., Zhai X.C., Zhang Y., Duan Z., Sun J.R. (2018). Optimization of enzyme assisted extraction of polysaccharides from pomegranate peel by response surface methodology and their anti-oxidant potential. Chin. Herb. Med..

[22] Bian C., Xie N., Chen F. (2010). Preparation of bioactive water-soluble pachyman hydrolyzed from sclerotial polysaccharides of *Poria cocos* by hydrolase. Polym. J..

[23] Yin X., You Q., Jiang Z. (2011). Optimization of enzyme assisted extraction of polysaccharides from *Tricholoma matsutake* by response surface methodology. Carbohyd. Polym..

[24] Gao M.J., Liu L.P., Li S., Lyu J.L., Jiang Y., Zhu L., Zhan X.B., Zheng Y.Y. (2020). Multi-stage glucose/pachymaran co-feeding enhanced endo-β-1,3-glucanase production by *Trichoderma harzianum* via simultaneous increases in cell concentration and inductive effect. Bioproc. Biosyst. Eng..

[25] Sánchez O.F., Rodriguez A.M., Silva E., Caicedo L.A. (2010). Sucrose biotransformation to fructooligosaccharides by *Aspergillus* sp.. N74 free cells. Food Bioprocess Technol..

[26] Ajila C.M., Gassara F., Brar S.K., Verma M., Tyagi R.D., Valéro J.R. (2012). Polyphenolic antioxidant mobilization in apple pomace by different methods of solid-state fermentation and evaluation of its antioxidant activity. Food Bioprocess Techol..

[27] Chai Y., Kan L., Zhao M. (2019). Enzymatic extraction optimization, anti-HBV and antioxidant activities of polysaccharides from *Viscum coloratum* (Kom.) *Nakai*. Int. J. Biol. Macromol..

[28] Ivan A.L.M., Campanini M.Z., Martinez R.M., Ferreira V.S., Steffen V.S., Vicentini F.T.M.C., Vilela F.M.P., Martins F.S., Zarpelon A.C., Cunha T.M. (2014). Pyrrolidine dithiocarbamate inhibits UVB-induced skin inflammation and oxidative stress in hairless mice and exhibits antioxidant activity in vitro. J. Photochem. Photobiol. B Biol..

[29] Zhang Z., Wang X., Yu S., Yin L., Zhao M., Han Z. (2011). Synthesized oversulfated and acetylated derivatives of polysaccharide extracted from *Enteromorpha linza* and their potential antioxidant activity. J. Ethnopharmacol..

[30] Wang Q., Chen S., Han L., Lian M., Wen Z., Jiayinaguli T., Liu L., Sun R., Cao Y. (2014). Antioxidant activity of carboxymethyl (1→3)-β-D-glucan (from the sclerotium of *Poria cocos*) sulfate (in vitro). Int. J. Biol. Macromol..

[31] Chen Y., Xie M.Y., Nie S.P., Li C., Wang Y.X. (2008). Purification, composition analysis and antioxidant activity of a polysaccharide from the fruiting bodies of *Ganoderma atrum*. Food Chem..

[32] Siddhuraju P., Becker K. (2007). The antioxidant and free radical scavenging activities of processed cowpea (*Vigna unguiculata* (L.) Walp.) seed extracts. Food Chem..

